# Charge Transport in Trap-Sensitized Infrared PbS Quantum-Dot-Based Photoconductors: Pros and Cons

**DOI:** 10.3390/nano8090677

**Published:** 2018-08-30

**Authors:** Alberto Maulu, Juan Navarro-Arenas, Pedro J. Rodríguez-Cantó, Juan F. Sánchez-Royo, Rafael Abargues, Isaac Suárez, Juan P. Martínez-Pastor

**Affiliations:** 1 UMDO, Instituto de Ciencia de los Materiales, Universidad de Valencia, P.O. Box 22085, 46071 Valencia Spain; albertomaulu@hotmail.it (A.M.); juan.navarro-arenas@uv.es (J.N.-A.); p.rodriguez@intenanomat.es (P.J.R.-C.); Juan.F.Sanchez@uv.es (J.F.S.-R.); rafael.abargues@uv.es (R.A.); isaac.suarez@uv.es (I.S.); 2 Intenanomat SL, Catedrático José Beltrán 2, 46980 Paterna, Spain

**Keywords:** PbS quantum dots, quantum dot solid, ligand exchange, solution processing, doctor blade, PbS QD photoconductivity, PbS QD photodetectors

## Abstract

Control of quantum-dot (QD) surface chemistry offers a direct approach for the tuning of charge-carrier dynamics in photoconductors based on strongly coupled QD solids. We investigate the effects of altering the surface chemistry of PbS QDs in such QD solids via ligand exchange using 3-mercaptopropionic acid (MPA) and tetrabutylammonium iodide (TBAI). The roll-to-roll compatible doctor-blade technique was used for the fabrication of the QD solid films as the photoactive component in photoconductors and field-effect phototransistors. The ligand exchange of the QD solid film with MPA yields superior device performance with higher photosensitivity and detectivity, which is due to less dark current and lower noise level as compared to ligand exchange with TBAI. In both cases, the mechanism responsible for photoconductivity is related to trap sensitization of the QD solid, in which traps are responsible of high photoconductive gain values, but slow response times under very low incident optical power (<1 pW). At medium–high incident optical powers (>100 pW), where traps are filled, both MPA- and TBAI-treated photodevices exhibit similar behavior, characterized by lower responsivity and faster response time, as limited by the mobility in the QD solid.

## 1. Introduction

During the last decade, lead-chalcogenide colloidal quantum dots (QDs) were shown to be attractive materials for new-generation optoelectronic devices due to the large tunability degree of their optical properties together with their solution processability. In this way, the application of lead-chalcogenide QD solids (the QDs are self-assembled forming a super-crystal) as thin films revealed outstanding potential applications in high-efficiency optoelectronics, such as solar cells [1,2], field-effect transistors [3], light-emitting diodes [4], and photodetectors [5,6]. Aiming for high optoelectronic performance of QD-solid-based devices, the engineering of the QD solids and the control of their chemistry were shown to be key factors [7,8].

The processing of QDs for the production of solid state films was explored through cheap, fast, and straightforward solvent-based deposition techniques. Methods such as drop casting, spin coating, and dip coating of colloidal nanocrystals are extensively and mostly used for the fabrication of devices, as reported in literature [9,10,11]. Regardless of the solution processing deposition method, the fabrication of compact and homogeneous QD solids with controlled thickness and low structural-defect concentration was addressed to develop high-efficiency photodetector devices [6,12,13]. Alongside the QD-solid fabrication technique, significant work was devoted to the study of the chemical composition of nanocrystal dispersion since it was shown to be a crucial factor in the optimization of charge-carrier dynamics in the QD solid [14].

In colloidal solutions, lead-chalcogenide QDs are commonly surrounded by long aliphatic ligands, which have a stabilizing and passivating role, allowing for flexible control over their size, shape, and composition [15] while preventing the formation of aggregates. Since ligands set the potential barrier for charge-carrier transfer and transport in QD solids, long insulating ligands must be replaced with shorter ones in order to enhance the coupling between QDs while still maintaining the carrier quantum confinement and efficiently passivating their surface [8,16,17]. Several ligand-exchange strategies were reported for lead-chalcogenide QDs, both in solution and in solid state, using bidentate thiols [18], primary amines [19], carboxylic acids [20], thiocyanate ions [21], and halide ions [2]. Each one of these ligands has different chemical properties and selective affinity for the nanocrystal facets, bringing distinctive behavior of the charge-carrier dynamics in the QD solids [22]. Also, the ligand-exchange procedure is relevant for charge-carrier dynamics in the QD solids, since surface defects in QDs can also be generated due to an incomplete ligand substitution or charge imbalance between the ligand and the surface states [16]. These surface defects in QDs are dependent on the length and the chemical structure of the capping ligands surrounding and interconnecting the QDs [23]. Furthermore, they introduce trapping states energetically located within the QD band gap that modify the photoconductivity kinetics [24]. On one hand, the presence of some kind of these intra-gap states has detrimental effects on optoelectronic devices; thus, great efforts were dedicated to their passivation [25,26]. On the other hand, all studies carried out on photoconductor detectors based on QD solids suggest that some other intra-gap states are acting as sensitizer centers (or safe traps) leading to very high photoconductive gain (G) [27,28]. This is because electrons can easily be captured after electron-hole photoexcitation, while holes remain relatively free to move through the QD solid. In any case, despite the available literature, the exact origin, the behavior, and the influence that intra-gap states have on the optoelectronic properties of QD solids are not yet fully understood [29,30]. In PbS QDs, the origin of intra-gap states was attributed to an off-stoichiometry Pb-rich surface [8] or to charge imbalance between Pb atoms and capping ligands [18]. Particularly, lead sulfates (PbSO_4_), formed at the surface of the QDs after air exposure, play an important role in the electrical properties of PbS QD solids. They can firstly act as p-dopant agents and trapping centers whose charge-carrier capture coefficient is about 400 times higher for electrons [28], also being traps responsible for the increase in minority carrier lifetime [24]. Such behavior can be detrimental for the application of these nanocrystals in solar cells or light-emitting diodes; however, in photoconductors, it gives rise to a high G-value under very low power excitation when an external electric field is applied [28]. This mechanism can lastly be a path for efficiently increasing the photoconductor detectivity; however, it also limits the temporal response of the device, which requires short carrier lifetime and fast charge-carrier collection. It is, thus, evident that these conditions establish a fundamental trade-off toward the achievement of high G and large bandwidth in photoconductors.

Here, we present the study of PbS QD-solid-based infrared photodetectors fabricated in ambient conditions using a simple and low-cost deposition technique. The device metal contacts were patterned by ultraviolet (UV) photolithography and lift-off processing prior to the PbS QD solid formation using the roll-to-roll compatible doctor-blade technique. We focused our study on the effects of two different post-processing solid-state ligand-exchange treatments using the bidentate aliphatic thiol, 3-mercaptopropionic acid (MPA), and the iodine-containing ligand, tetrabutylammonium iodide (TBAI). The interest in approaching these two passivation strategies arises from the understanding of how their different chemical natures influence the QD-solid properties and the effects on the electro-optical properties of the device. The use of ligand exchange by MPA, which has both thiol and carboxylate functional groups, provides a high passivation action over a broad distribution of PbS surface states, simultaneously enabling high electronic coupling among QDs (enhanced electronic wave-function overlap) [16]. Instead, the post-processing treatment with TBAI was approached aiming to further enhance the QD coupling by introducing I^−^ ions that bind to Pb^2+^ dangling bonds at the PbS surface [31]. Additionally, the formation of PbI_2_ at the surface with an I^−^:Pb^2+^ ratio of 1:1 can result in the formation of a PbI_2_ shell-like structure around the PbS core, which can be considered as a passivating action [32]. We finally compare TBAI, to achieve short interparticle distances, with MPA, which likely would lead to a more complete passivation of the QD surface defects [16], in order to define how these different strategies influence the optoelectronic performances of QD-solid-based photoconductors. Our results, based on the correlation between the electronic properties of the film deduced from X-ray photoelectron spectroscopy (XPS) measurements and the electro-optical properties of the photoconductor devices, confirm that MPA-treated films led to superior responsivity (~80 A/W at 1550 nm), due to very low conductivities under dark conditions (low free-carrier concentration or doping), and higher detectivities (D* ~ (1–5) × 10^12^ Jones). Contrary to the aprioristic assumption expressed above, TBAI-treated QD solids led to higher dark conductivities, limiting the sensitivity and detectivity of photodevices. This was ascribed to an additional charge-transport mechanism related to mid-gap states, even if mobility was significantly lower than in MPA-treated films. We modeled the photocurrent (and responsivity) by including the excess of minority carriers photogenerated by absorption and the presence of a single trap level, which nicely reproduced the observed evolution with light power received by the photoconductors, both MPA- and TBAI-treated. This modeling explains the high observed responsivities under very low incident powers (if photocurrent is superior to noise current) because most photogenerated carriers were trapped (usually known as sensitization of the photoconductors), whereas free minority carriers were responsible for photocurrent/photoconductivity in the QD solid.

## 2. Experimental Section

### 2.1. Materials

Sulfur (99.999%, S), lead chloride (99.999%, PbCl_2_), 3-mercaptopropionic acid (99%, MPA), and tetrabutylammonium iodide (98%, TBAI) were purchased from Sigma-Aldrich (Madrid, Spain) and used as received. Oleylamine (OAm), methanol, and octane were all synthesis-grade quality.

### 2.2. PbS Quantum-Dot Synthesis

PbS QDs were synthesized using a modification of the protocol described by Cademartiri et al. [33]. In this synthesis, lead chloride (PbCl_2_) and elemental sulfur (S) are the precursors, and oleylamine (OAm) is the coordinating solvent, which simultaneously acts as a ligand and a solvent. Firstly, a solution of 0.05 g of S (1.5 mmol) in 5 mL of OAm was prepared in a round-bottom flask, heating the mixture under N_2_ at 125 °C for 60 min. Meanwhile, 1.4 g of PbCl_2_ (5 mmol) and 15 mL of OAm were mixed and degassed under N_2_ in a three-neck flask at 125 °C for 60 min, resulting in a turbid white suspension. The lead oleate dispersion was then heated up to 140 °C, followed by the swift injection of the S–OAm precursor solution. The solution turned dark brown over the course of a few seconds. The reaction time was set to 20 min to obtain the required QD size. Afterward, the solution was allowed to slowly cool.

The obtained dispersion was then isolated from the unreacted precursors by adding 15 mL of toluene, before being centrifuged. Further purification was performed by adding 5 mL of methanol and centrifuging to precipitate secondary products and the excess ligand. We then solvated and precipitated the PbS QDs again following the same process. At this point, the QD ink used for the doctor blade was made by solvating the final precipitation in octane at a concentration of 150 mg/mL.

### 2.3. PbS QD-Solid Deposition Using the Doctor-Blade Technique

The QD solids were fabricated by processing the QD ink described above via the doctor-blade deposition technique using a motorized film applicator (Elcometer 4340, Elcometer Limited, Manchester, UK). The distance between the coating blade and the substrate was fixed at 1 mm for the injection of 4 μL of PbS nanoink, followed by the ink spreading at 1.5 cm·s^−1^ over three microgap electrodes (a total area of around 1 mm × 2 cm was covered) to create an initial thin (30 nm) coating of PbS QDs. The initial film was deposited at the indicated velocity (1.5 cm·s^−1^) in order to create a “buffer QD layer”, which was followed by faster spreading cycles at 6 cm·s^−1^ to obtain thicker QD layers, of ~90 nm each, until the desired thickness was achieved (total thickness of around 300 nm). The ink was always spread in the forward direction, and the blade was recharged again with ink for subsequent cycles. A baking process of 5 min at 100 °C was performed after every deposition, whereas a thermal curing process was carried out at 100 °C for 90 min under vacuum conditions. We produced QD solids using the doctor-blade approach onto silicon–SiO_2_ substrates with gold electrodes (80 nm thick with interelectrode gaps of 2, 5, and 20 μm) for the realization of photoconductor devices. The electrode patterns were previously prepared using UV photolithography and lift-off processing, and the metal deposition was carried out using thermal evaporation under vacuum at a rate of 1 Å/s.

For absorption and XPS measurements (and C–V curves carried out in Schottky-heterojunction diodes), the doctor-blade method was applied to cover glass substrates (and glass/ITO/PEDOT) of around 1 × 1 square inches of area, as described previously in Reference [5]. The Schottky-heterojunction diodes were only used in the present work to measure C–V curves (data included in the Supplementary Materials), because MPA-based devices were previously studied as photodetectors in Reference [5].

### 2.4. Solid-State Ligand Exchange

The post-deposition solid-state ligand exchange was carried out following a previously reported procedure [5]. The ligand-exchange solutions were prepared by dissolving the ligands in methanol at a concentration of 10 mM, before dipping the OAm-capped PbS QD solids into the obtained solution for 60 s. The treated films were rinsed in methanol to remove the ligand excess, before being dried under N_2_ stream and thermally cured under vacuum for 90 min at 100 °C.

### 2.5. Structural and Electronic Characterization of PbS QDs and QD Solids 

The morphology of the nanocrystals and their size dispersion were evaluated with a JEOL JEM-1010 transmission electron microscope (JEOL Ltd, Tokyo, Japan) at 100 kV. Cross-sectional scanning electron microscopy (SEM) was performed using a Hitachi 4800 microscope (Hitachi High-Technologies Corporation, Tokyo, Japan) at 10 kV of acceleration voltage. X-ray photoelectron spectroscopy (XPS) measurements were performed in an ultra-high vacuum system (base pressure 1.0 × 10^−10^ mbar), ESCALAB-210 (Thermo Scientific, Waltham, MA, USA). Photo-electrons were excited using the Mg Kα line (1253.6 eV). The C 1s peak was used as the reference for the binding energy (fixed to 285 eV).

QD-solid infrared absorption measurements were performed using a home-built set-up. The excitation source consisted of a halogen lamp (20 W) focused into a multimode optical fiber that was coupled to a DeltaNu DNS-300 monochromator (Intevac Inc., Santa Clara, CA, USA) (grating of 600 g/mm blazed at 1200 nm). A Newport DET-L-GE-T-C calibrated Ge photodetector (Newport Corporation, Irvine, CA, USA) was used to quantify the light intensity ratio with/without sample (transmittance).

### 2.6. Electrical Characterization of Photodevices

Devices I–V were tested under ambient conditions at room temperature using a white halogen lamp as the illumination source, with intensity adjusted to 250 W/m^2^. The curves were recorded using a Keithley Series 2400 Source Meter Unit (Keithley Instruments Inc., Cleveland, OH, USA). Light intensity was measured using a calibrated Si solar cell. Photoconductors were mounted on a home-built sample holder designed to homogeneously distribute light across the photodetector active area.

The PbS QD solids were deposited and formed as described above (Section 2.3 and Section 2.4) over prefabricated OFET (Organic Field Effect Transistor) test chips from Ossila (Sheffield, UK), and were mounted for characterization on a high-density OFET test board (also from Ossila), designed to reduce external noise, leakage current, and stray capacitance. Drain-source current was measured at 1-V bias using the Keithley SMU, and the gate bias was applied using a DC power source connected to the BNC connector board. All measurements were carried out using the LabView automated test suite (National Instruments SL, Madrid, Spain). We also fabricated simple Schottky-like diodes (glass/ITO/PEDOT/QD solid/Ag), where the QD solids (MPA and TBAI treated) were produced using the same fabrication process and conditions carried out for the photoconductors. The capacitance-versus-voltage curves of these diodes were registered using an IET/Quadtech 1920 precision LCR meter (IET Labs Inc., Roslyn Heights, NY, USA).

### 2.7. Electro-Optical Characterization of Photodevices

The optoelectronic performances of the photodetector in the infrared region (>1100 nm) were measured using a home-built set-up. The excitation source and monochromator were the same as those used in the transmittance measurements of the QD films. The light was mechanically chopped at the frequency of interest before being focused through the glass substrate to the photoconductor active surface using a 10× objective. The device photocurrent was measured using a SR810 DSP lock-in amplifier (Stanford Research Systems, Sunnyvale, CA, USA). The illumination intensity was varied by tuning the DC voltage applied to the tungsten lamp. The optical power impinging on the active area of the detector was measured by means of the same calibrated Ge photodetector used in transmittance experiments. A programmable voltage source, Keithley 230 (Keithley Instruments Inc., Cleveland, OH, USA), was used to apply a voltage bias to the device, connected in parallel with a load resistor of 1 MΩ and a capacitor of 0.1 μϜ in series with the lock-in amplifier, so as to cut the DC current. The noise current used for the experimental determination of the photoconductor noise equivalent power (NEP) [5] was measured under dark conditions at different integration times using the internal sine function provided by the lock-in amplifier. For the transient photocurrent measurements, the biased photoconductor devices were illuminated with a 20-KHz ns-pulsed laser at 1064 nm and the signal (coupled through a 50-Ω termination) was monitored using a digital 100-MHz oscilloscope.

## 3. Results and Discussion

The fabrication of PbS QD-solid-based photoconductor detectors was carried out via the solution processing of a PbS nanoink formulated for the doctor-blade technique. This nanoink exhibited a surface tension of 36 dyn·cm^−1^ and a contact angle of ~6° onto the SiO_2_/Si substrates.

**Figure 1:**
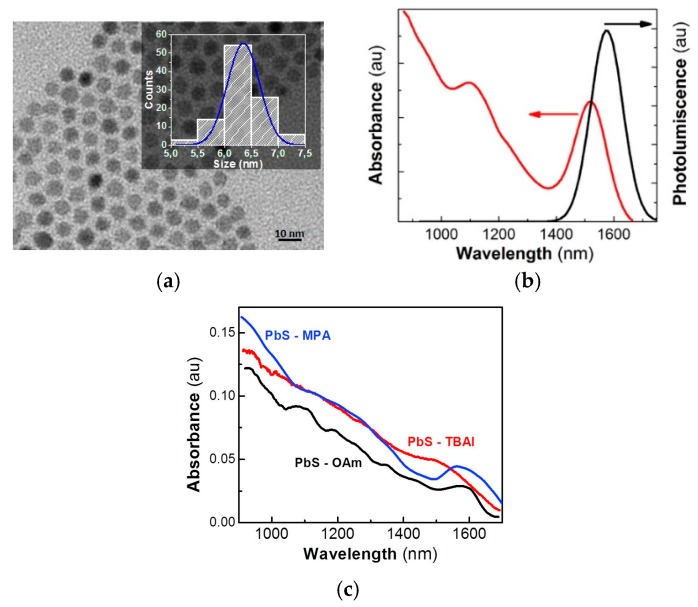
(**a**) Transmission electron microscopy image of PbS quantum dots (QDs) and size histogram (inset). (**b**) Absorbance and photoluminescence spectra measured in the QD nanoinks used to create films with the doctor-blade technique. (**c**) Absorbance spectra of PbS QD solids measured (a finite background absorbance due to scattering was subtracted from the raw spectra) in untreated (black) and after post-deposition ligand exchange with tetrabutylammonium iodide (TBAI; red) and 3-mercaptopropionic acid (MPA; blue).

The PbS QDs had an average diameter of 6.4 nm with a size dispersion of ~9% (Figure 1a), and showed excitonic absorption and photoluminescence resonances centered at 1520 and 1575 nm, respectively, with a bandwidth of 140 nm, as observed in Figure 1b. The 300-nm-thick PbS QD solids were fabricated using the doctor-blade technique, and the ligand-exchange process was carried out with TBAI and MPA after film formation. High-quality homogeneous films were obtained. Neither cracks nor pinholes appeared in the QD solids after the ligand exchange, as shown in the cross-sectional SEM image in Figure S1, and as previously reported by us and other authors [5,34]. In Figure 1c, we can observe the influence of the ligand exchange on the optical properties of the PbS QD solids. Their exciton absorption resonance can be clearly observed very close to 1600 nm before and after MPA ligand exchange. However, the TBAI-treated film shows a more important broadening of the excitonic band. Given that inter-particle spacing among PbS QDs decreased dramatically, QDs probably merged into bigger PbS particles (or agglomerates), losing part of their quantum confinement characteristics [8]. On the other hand, this effect was not observed (or negligible) in the MPA-treated PbS solids, which, in turn, allowed the maintenance of the quantum confinement properties while keeping short interparticle distances, and hence, leading to a more homogeneous QD solid (Figure S2) with electronic coupling between QDs.

**Figure 2:**
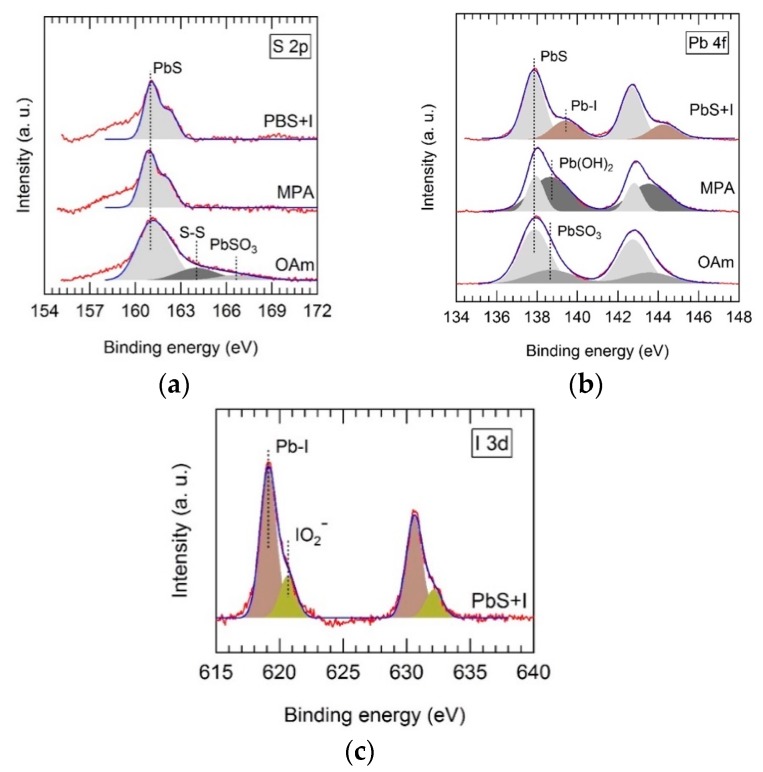
X-ray photoelectron spectroscopy (XPS) spectra of the PbS QD solid treated with TBAI and MPA compared to the untreated film in the energy regions of S 2p (**a**) and Pb 4f (**b**) photoelectron transitions; (**c**) I 3d XPS spectrum of the PbS QD solid treated with TBAI.

Ligand exchange of OAm-capped PbS QD solids with TBAI and MPA was followed using XPS. Depending on the ligand used, we observed strong changes in the chemical state of atoms at the surface of PbS QDs. The XPS spectra of PbS-OAm QD solids appear to be dominated by spin-orbit doublets, whose S 2p_3/2_ and Pb 4f_7/2_ components were located at 160.9 ± 0.2 and 137.9 ± 0.2 eV, as shown in Figure 2a,b, respectively. These transitions can be attributed to bulk-like PbS. Their corresponding XPS intensities show a S:Pb ratio of 1.4:1, which is reasonably close to unity [35]. Moreover, we observed weaker components at 164.0 and 166.6 eV, which can be attributed to S–S bonds and PbSO_3_ species located at the surface of the PbS QDs. These species were suppressed after the ligand exchange with both MPA and TBAI, as shown in Figure 2a. However, MPA-capped PbS shows a strong Pb 4f_7/2_ component located at 138.7 eV (Figure 2b), probably due to the presence of Pb(OH)_2_ species at the surface of the QDs. This component was strongly reduced in TBAI–PbS where a new Pb 4f doublet can be observed (Figure 2b), and can be attributed to Pb–I bond formation at the QD surface. Furthermore, in TBAI-treated PbS QD solids, we can observe an intense I 3d photoelectron doublet, whose *j* = 5/2 component appears at 619.1 eV, as shown in Figure 2c (brown-shaded peaks). The presence of this peak can be attributed to I bound to Pb at the QD surface with an I:Pb ratio of 1.2:1. At higher energies, a weaker I 3d photoelectron doublet can be observed, whose *j* = 5/2 component is located at 620.6 eV (green-shaded peaks in Figure 2c). This can be ascribed to residual I atoms forming IO_2_^−^ ions, which, in turn, prevent the formation of lead oxidative species, as noted above.

After XPS analysis, one can conclude that QD solid films result in very different QD solid compositions depending on the ligand used. Before ligand exchange, PbS–OAm solids showed oxidation species such as PbSO_3_ at the surface of PbS QDs from the oxidation of S^2−^. After ligand exchange, no oxidation of S^2−^ was observed, but new species were formed such as Pb–OH in MPA–PbS, and Pb–I and IO_2_^−^ in TBAI–PbS. The influence of MPA and TBAI on the electro-optical properties of the PbS-films is thoroughly discussed below.

**Figure 3:**
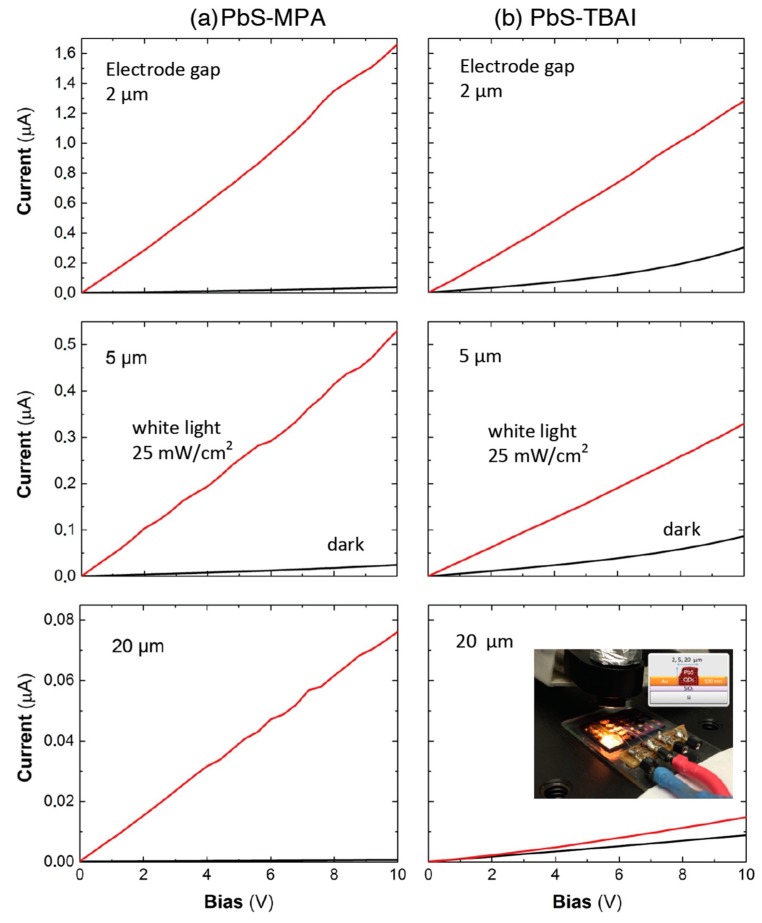
Current–voltage characteristics in the dark (black lines) and under 25-mW/cm^2^ halogen-lamp illumination (red lines) of processed PbS colloidal QD photoconductors: MPA-treated (**a**), and TBAI-treated (**b**). The inset in the bottom-right panel shows the three contacted photodevices under white-light illumination (the picture also includes a scheme of the photoconductor detector).

Figure 3 shows the current-versus-voltage (0–10 V) curves in the dark and under white-light illumination for photoconductor devices based on PbS QD solids (see the inset in the bottom-right panel) after ligand exchange with MPA (left panels) and TBAI (right panels), presenting various inter-electrode gaps (indicated in the plots). All the electrical/photo-electrical parameters extracted from these curves are listed in Table 1. Clearly, MPA-treated PbS QD layers (panels in Figure 3a) display smaller dark-current levels (black curves) and larger photocurrents (red curves) than TBAI-treated ones (panels in Figure 3b). Such differences were translated into photo-sensitivities, defined as *S =* *(I_L_ − I_D_)/I_L_* *=* Δ*σ/(σ*_0_ *+* Δ*σ)* (*I_L_* and *I_D_* are the currents measured under illumination and dark conditions at a given voltage, and σ_0_ and Δσ are the QD-solid conductivity and photoconductivity, respectively), which were very close to the unit in the first case (MPA-treated photodevices), as listed in Table 1.

The dark current was not linear in the whole range of applied voltages (0–10 V), more evident in the case of TBAI-treated samples for 2- and 5-μm-wide electrode gaps (see Figure 3b), which was the reason for variation in the dark conductivity *σ*_0_ (see note in Table 1). This nonlinear behavior (see double logarithmic plot in Figure S3 and a more complete analysis in the Supplementary Materials) can be attributed to the space-charge-limited current (SCLC) effect that occurs when uncompensated charge carriers are injected into the material, which is amplified by the presence of traps, as reported for Si-nanocrystal films [36]. The conductivity values estimated in the case of the photoconductor devices with an electrode gap of 20 μm (mostly for MPA-treated QD solids) were smaller than those obtained in the case of devices with electrode gaps of 2 and 5 μm, possibly due to the resulting thickness of the QD-solid film being smaller in the first case. On average, the conductivity measured in the QD solid through 2–5-μm-wide electrodes was *σ_0_(MPA)* ~ 0.4 μS/cm after MPA treatment, and increased by a factor six, *σ_0_(TBAI)* ~ 2.5 μS/cm, in the case of TBAI (despite the absolute values, a similar increase in conductivity from MPA to TBAI processing was observed for the 20-μm-wide electrode). This can be explained by the equation, *σ_0_ = q μ_p_ p*, where *μ_p_* and *p* are the hole mobility and concentration, respectively, assuming that the QD solid was p-doped, particularly after MPA treatment [37] and exposure to ambient conditions [38,39]. Therefore, the smaller dark conductivity in MPA-treated films can be attributed to smaller values of one or both magnitudes as compared to TBAI-treated ones.

The small dark conductivities and very low hole mobility of the QD-solid films make it very difficult to achieve precise Hall-effect measurements; hence, two independent estimations were made in the present work. The first one is related to the hole mobility measured in FET devices, and the second one is the acceptor concentration deduced from the slope of C^−2^(V) curves measured in Schottky-like ITO/PEDOT/QD solid/Ag devices, as described in the experimental section in both cases. The curves of drain current versus gate voltage in FET devices and C^−2^(V) in Schottky photodiodes are shown in Figures S4 and S5, respectively, from which the effective mobility and acceptor concentration could be estimated for MPA- and TBAI-treated QD solids, as listed in Table 2. The concentration of acceptor impurities estimated for TBAI-treated films was one order of magnitude higher than that for MPA; however, mobilities were significantly lower, possibly due to an effect of nanocrystal agglomeration, as suggested above, instead of an average smaller inter-particle distance. From these values, the conductivity deduced for MPA-treated QD-solid films (Table 2), within the spread found in the hole mobility, (1–4) × 10^−4^ cm^2^/Vs (values reported in Reference [34] and [38,39] are within this interval), is consistent with values deduced from the I–V curves of the photoconductor devices (Table 1). A greater difference was observed in the estimation of dark conductivity for TBAI-treated QD-solid films, 0.07–0.22 μS/cm (Table 2), as compared to those measured from I–V curves of photoconductors, from 0.58 up to 4 μS/cm (Table 1), which can be attributed to a noticeable contribution to charge transport of a weakly conducting mid-gap energy band formed from deep levels originated at the surface of the PbS QDs after formation of the QD solid [40]. In this sense, the low built-in potential barrier, *V_bi_* ~ 0.15 V, deduced from C^−2^(V) curves in the Schottky photodiodes (Figure S5), is also consistent with *V_OC_* values (~0.1–0.3 V, see Figure S6), as deduced from I–V curves under AM1 illumination in these diodes. Such low *V_bi_* and *V_OC_* values are a signature of the presence of mid-gap states in both TBAI- and MPA-treated QD-solid films. However, in the latter case, the charge transport seems mainly due to free holes in the valence miniband of the QD solid, as formed from the three-dimensional electronic coupling of QDs, which are spatially ordered as a BCC (Body Centered Cubic) lattice [5].

**Figure 4:**
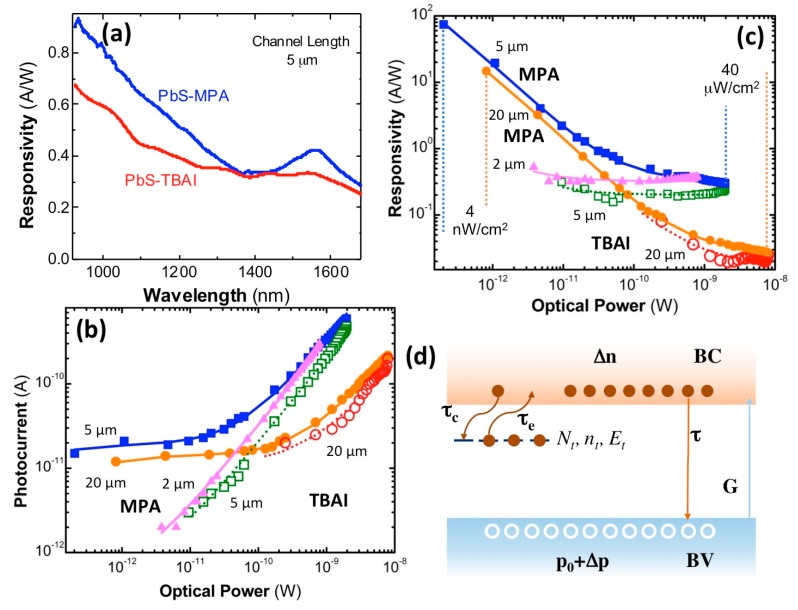
(**a**) Responsivity spectra of 5-μm-gap photoconductors under 100-V bias for PbS–MPA (blue) and PbS–TBAI (red) films. (**b**) Experimental (symbols) and calculated (lines) photocurrent of MPA- (solid symbols) and TBAI-treated (hollow symbols) photodevices as a function of optical power received (electrode gap width is indicated in the plot) at 1550 nm and 100 V of voltage bias (50 V for 2-μm-wide electrode gap). (**c**) The same as (**b**) for responsivity; the measured range for power density is indicated. (**d**) Illustration of the kinetic model for minority-carrier recombination including the presence of photoconductive sensitized centers (safe traps).

The responsivity curves in Figure 4a exhibit a very similar wavelength dependence to that observed in the corresponding absorbance spectra reported in Figure 1c, as expected if the photocurrent was proportional to the absorption coefficient spectrum of the material (if diffusion and surface recombination does not have an important effect). Again, the exciton resonance is much less pronounced in the TBAI-treated photoconductor device, as discussed above for absorption spectra in Figure 1c. The photoconductors (electrodes with 5 μm of channel length) exhibit peak values of around 0.45 and 0.35 A/W at the exciton resonance (~1550 nm) for MPA and TBAI treatments, whereas an increase for shorter wavelengths occurred due to the larger absorption coefficient of the PbS QD solid, up to 0.9 and 0.7 A/W at 950 nm, respectively. It is worth noting here that the spectral dependence of responsivity was measured at the maximum optical power of the halogen lamp (power density of ~40 μW/cm^2^ at the exit slit of the monochromator at 1550 nm, i.e., ~2 nW captured by the photodevice with 5 μm of channel length). The effect of the incident optical power on photocurrent (Figure 4b) and responsivity (Figure 4c) is discussed below on the basis of the safe-trap model illustrated in Figure 4d.

Figure 4b shows the power dependence of photocurrent measured at around the exciton resonance, 1550 nm, using a bias voltage of 100 V for all photodevices, except in the case of the 2-μm-wide electrode gap, where the photocurrent above 50–60 V saturates because of the sweep-out effect of minority carriers (electrons) [41] under such a large electric field (>200 kV/cm), for which the transit time, on the order of 1 μs for this electrode, is comparable to their lifetime. The photocurrent increases over the entire range of incident power, but much faster above ~20 pW in the case of photoconductors with 5-μm-wide electrode gaps (above ~100 pW in the case of the 20-μm ones). This behavior would be consistent with the expected linear power evolution of the photocurrent, or similarly, proportional to *gτ*, where *g* is the carrier generation rate and *τ*, the minority carrier lifetime, from the simplest generation/recombination model for photogenerated minority carriers (electrons). However, below those powers, the photocurrent practically does not vary significantly, which cannot be reproduced with such a simple model, but it is the origin of the huge increase in responsivity at very low powers, as observed in Figure 4c. In the case of the MPA-treated photoconductor with 2-μm-wide channel length and the 5-μm TBAI-treated one, we were not able to observe the photocurrent saturation effect at very low optical powers; however, here, the dark and noise currents were more relevant, and hence, it was near impossible to measure very low photocurrents (using 1–3 s of integration time in the lock-in amplifier). Correspondingly, the two optical-power regimes described for photocurrent were translated into a sharp decrease of responsivity by increasing the incident optical power and a saturation regime above such indicated powers.

The great responsivity values at very low incident powers and the subsequent fast decrease in power is typically attributed to trap-assisted sensitization of photoconductivity in semiconductors, and particularly, in PbS QD solids [42]. One of the best-measured values in our MPA-treated PbS QD-solid photoconductors reached ~80 A/W (photodevice with 5 μm of channel length) under 200 fW of incident monochromatic light at 1550 nm. Given the spectral dependence depicted in Figure 4a, the responsivity at 950 nm would be a factor twice as big as those measured at 1550 nm (~160 A/W). Therefore, the responsivities measured in MPA-treated photodevices were among the best values reported in the literature at the near-infrared region (900–1600 nm), as summarized in Table S1 of the Supplementary Materials, where some of the most important results collected in Reference [28] and more recent data are listed.

The lower responsivity measured in TBAI-treated photodevices at 1550 nm is a consequence of their larger dark current, as also reflected by their overall (white-light illumination) lower photo-sensitivities (Table 1) as compared to MPA-treated photoconductors. The TBAI treatment of QD solids can induce the introduction of one energetically dominant trap-state level at 0.34 eV below the conduction band of PbS, as pointed out in recent publications [25,43]. This mid-gap band level and similar ones for MPA-treated photodevices, even if associated with different surface species as deduced from XPS measurements, could be responsible for the QD-solid photoconductivity when not completely populated (trap-sensitized semiconductor) [42], i.e., under very low illumination intensities. Moreover, the effect of QD agglomeration and the expected strong electronic coupling in TBAI-treated PbS QD solids could also convert such a trap-state level into an energy band responsible for the higher dark-current levels in comparison to MPA-treated ones, as discussed above. Another consequence of this effect is a higher noise signal, influencing the minimum measurable photocurrent under very low illumination intensities (below 2–3 pW for 5-μm-wide-channel photoconductors).

As discussed above, photoconductors based on PbS QD solids are characterized by very large responsivities or very high photoconductive gain, but only at extremely low incident optical powers. Moreover, such a high photoconductive gain has its origin in the presence of trap states for electrons (the minority carriers in the PbS QD solid) close to the conduction band, i.e., centers that do not emit/capture holes, as illustrated in Figure 4d. From this model, by assuming the charge neutrality condition, $\Delta p=\Delta n+n_{t}$, where Δn (Δp) and $n_{t}$ are the excess photogenerated electron (hole) and occupied trap concentrations, respectively, the rate equations for minority carriers and trap states are as follows [44]:

(1) $$\frac{d\Delta n}{dt}=g-\frac{d\Delta n}{\tau }+\frac{n_{t}}{\tau_{e}}-\frac{\Delta n}{\tau_{c}}\left(1-\frac{n_{t}}{N_{t}}\right),$$

(2) $$\frac{dn_{t}}{dt}=-\frac{n_{t}}{\tau_{e}}+\frac{\Delta n}{\tau_{c}}\left(1-\frac{n_{t}}{N_{t}}\right),$$

where *τ*_c_ (*τ*_e_) is the capture (emission) time of electrons by (from) the traps whose total concentration is *N_t_* (and energy depth *E_t_*). The equations can readily be solved under steady-state conditions, *d*Δ*n/dt = dn_t_/dt = 0*, to obtain the following equations for Δ*n* and *n_t_*:

(3) $$\Delta n=g\tau ,\;n_{t}=\frac{g\tau \;N_{t}}{g\tau +N_{t}\left(\frac{\tau_{c}}{\tau_{e}}\right)},$$

from which the photoconductivity results in the following equation:

(4) $$\Delta \sigma =q\left(\mu_{n}+\mu_{p}\right)\Delta n+q\mu_{p}n_{t}=q\mu_{p}\left[\left(1+\frac{\mu_{n}}{\mu_{p}}\right)+\frac{N_{t}}{g\tau +N_{t}\left(\frac{\tau_{c}}{\tau_{e}}\right)}\right]g\tau .$$

From Equation (4), we clearly obtain the limits (i) $\Delta \sigma \approx q\mu_{p}\left(\frac{\tau_{e}}{\tau_{c}}\right)g\tau$ for very low carrier-generation level, $g\tau \ll N_{t}\left(\frac{\tau_{c}}{\tau_{e}}\right)$, and (ii) $\Delta \sigma \approx q\left(\mu_{n}+\mu_{p}\right)g\tau$ for high carrier-generation levels, $g\tau \gg N_{t}\left(\frac{\tau_{c}}{\tau_{e}}\right)$. Approximation (i) leads to very high photoconductivity gain and responsivity, provided $\frac{\tau_{e}}{\tau_{c}}\gg 1$, i.e., very low (thermal) emission of electrons from trap states to the conduction band. On the other hand, approximation (ii) explains the saturation of photoconductor responsivity for high incident optical powers, i.e., where photocurrent is proportional to the incident optical flux, the only regime for photodiodes [5]. The photocurrent and responsivity dependence with the incident optical power are nicely accounted for by Equation (4) (multiplied by the applied electric field and the photodevice surface, and expressing *g* as a function of the absorbed optical power), whose best fits are shown by continuous lines in Figure 4b,c for MPA- (solid lines) and TBAI-treated (dotted lines) photoconductors. From these fits (the most representative is the one for the 5-μm-channel photoconductor with MPA treatment), we obtain trap concentrations of ~10^11^ cm^−3^, *τ_e_/τ_c_* as ~2000–6000, and *τ/τ_d_* as ~0.05, where *τ_d_ = L^2^/μV* (*L* is the photoconductor channel length, *μ* is the effective carrier mobility, and *V* is the applied voltage) is the drift/transit time for minority carriers. If we use *τ_d_* as ~10 μs on the basis of the measured mobility (Table 2), the minority-carrier lifetime would result in ~0.5 μs, which is not far from values measured in the literature for colloidal PbS QDs and QD solids [34,43]. On the other hand, the ratio, *τ_e_/τ_c_*, should be due to the thermal exponential factor related to *E_t_*; hence, the energy depth of the trap level becomes in the range 195–225 meV, consistent with values estimated for trap states in PbS QD solids [25,30,43,45,46].

**Figure 5:**
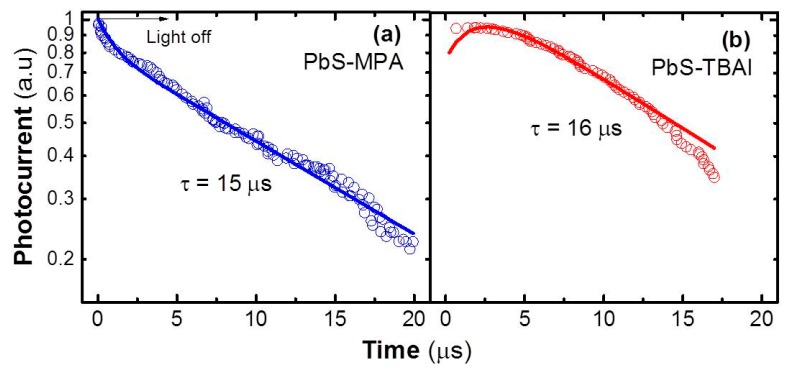
Transient photocurrent measurements for (**a**) MPA- and (**b**) TBAI-treated PbS QD-solid photoconductors with 5-mm channel length under pulsed laser excitation at 1064 nm.

As mentioned previously, the high responsivity regime of PbS QD solid photoconductors is simultaneously accompanied by a slow operation speed of photoconductors due to the term $\left(\frac{\tau_{e}}{\tau_{c}}\right)\tau$ under very low incident optical powers. From our estimate, the expected response time $\left(\frac{\tau_{e}}{\tau_{c}}\right)\tau$ is ~1–3 ms, which is consistent with reported values under low optical excitation densities [23,46]. On the contrary, when increasing the incident optical power, one would expect a decrease in the photodevice response time, by increasing the carrier-generation level. Indeed, the transient photocurrent signals measured under relatively high power using an ns-pulsed laser at 1064 nm, where free minority carriers dominate and responsivity tends to saturate, exhibit a decay with time constants on the order of some tens of μs, as shown in Figure 5, where the rise time is practically negligible (<1 μs) within the time resolution of the oscilloscope used in the experiment. Particularly in the case of the MPA-treated photodetector, the photocurrent signal exhibits a main decay time of ~15 μs (Figure 5a) after a first fast decay <3 μs (the fit was made using these two decay times and a rise time <1 μs), which is consistent with the effective recombination time of minority carriers measured under high incident optical power in solar cells based on PbS QD solids after MPA ligand exchange [23]. In the case of the TBAI-treated photoconductor, there is only one dominant component with a time constant of ~16 μs (Figure 5b) without the initial fast decay observed in the MPA-treated photodevice (the fit was made using this single decay and a rise time of ~2 μs). Both decay times, 15–16 μs, are not far from the drift/transit times expected in these QD solids (Table 2) at the bias voltage used in the experiment (100 V), because it is carried out under high incident light power where most of the traps are filled. Under these conditions, it is also important to highlight that the response time of the photoconductors is faster than that obtained in Schottky-based photodiodes (both treated with MPA), where a value of ~135 μs was measured [5].

The expression for calculating detectivity at a given light wavelength and bias voltage can be written as a function of the dark current in the photoconductor if shot noise dominates, as recently used in Reference [47] to estimate detectivity in organic/PbS bilayers.

(5) $$D^{*}=R_{\lambda }\sqrt{\frac{S}{2qI_{dark}}},$$

where *R_λ_* is the responsivity at the wavelength of *λ* (1550 nm in our case), *S* is the effective area of the photoconductor (*S = L* × *W*, where *L* and *W* are the channel length and width, 2–5–20 μm and 1 mm, respectively), and *q* is the absolute value of electron charge. The best values for detectivity were found for MPA, as expected from previous discussions; concretely, values were ~10^12^ Jones (*I_dark_* (100 V) ~1 μA) for the photoconductor with 5-μm channel length, and ~5 × 10^12^ Jones in the case of the 20-μm one (*I_dark_* (100 V) ~6 nA), whereas it was below 10^10^ Jones for the 2-μm one because of the smaller area and higher dark current (0.45 μA at 50 V). Again, D* values at 1550 nm were among the best-reported values for photoconductor devices based on PbS QD solids, particularly in those using MPA ligand exchange (see Table S1). For the corresponding photodevices using TBAI treatment, we obtained ~1.7 × 10^9^ and ~5 × 10^10^ Jones, again because of the high dark currents for these photodevices (150 nA and 5.4 μA at 100 V for 5- and 20-μm-wide channel lengths, respectively), as discussed above.

It is also important to highlight here that the high detectivities deduced in MPA-treated photodevices by Equation (5) only occur under very low incident light due to the presence of sensitizing trap centers for minority carriers, which produces a saturation of the photocurrent value very close to the noise current. Furthermore, it was reported recently that 1/*f* noise can be more important in PbS QD photoconductors than shot noise [48,49]. For this reason, the following more general expression would be more accurate for estimating D*:

(6) $$D^{*}=\frac{\sqrt{S\Delta f}}{NEP},$$

where *NEP* is the noise equivalent power that can be estimated experimentally from photocurrent as a function of incident power and extrapolation to noise current value, which in our case is ~10 pA within a bandwidth of 1.6 Hz in the photodevice with 5-μm channel length, very close to the smallest measured photocurrent value at the lowest incident power (100 fW) that would imply D* of >10^11^ Jones. However, this experimental determination of NEP is not exempt from inaccuracy due to the saturation effect of photocurrent in the low incident power range in PbS QD-solid photoconductors (MPA-treated ones, mainly), as discussed above. This is not the case for the other photoconductors where trap sensitization is not effective over noise (trap-level densities < 10^11^ cm^−3^), which also occurs in photodiodes where short-circuit current always varies linearly with incident power [5].

A final question to be considered for photoconductors, including those based on PbS QD solids, is the high bias voltages (20–100 V, depending on the channel length) used to get large responsivities. The solution to this issue is the use of interdigitated photoconductor devices, because smaller bias voltages are needed in a factor approximately equal to the number of metal finger pairs, for which promising results were obtained (see also Table S1) [48,50,51]. We also fabricated some previous generations of interdigitated photoconductors (see Figure S7) that reached a responsivity of around 7 mA/W at 10 V, which is not far from the value reported in Reference [48] for the same finger distance (20 μm). However, this value cannot be compared with the new generation of optimized two-electrode-gap photoconductors whose results are presented in this work, for which the doctor-blade deposition and thickness were optimized. In interdigitated photoconductors, our doctor-blade technique produces more inhomogeneous PbS QD-solid films over the interdigitated area, and further optimization (or a different deposition technique, as the one used in Reference [51]) is needed to develop efficient photodetectors operating at low voltages in the future.

## 4. Conclusions

The doctor-blade deposition technique was successfully applied for the fabrication of high-quality PbS QD-solid-based photoconductors with different inter-electrode channel lengths. We studied and compared the effect of TBAI and MPA post-processing solid-state ligand exchange on the optoelectronic properties of PbS QD-solid-based photoconductors. We achieved the best overall photoconductive figures of merit using MPA, mainly attributed to the different chemical nature of passivation of the PbS QD surface defects (Pb–OH bonds), as compared to TBAI (Pb–I bonds). In the first case, the charge transport was mainly due to majority carriers (holes) through the valence miniband formed in the QD solid by the three-dimensional electronic coupling between QDs. The short inter-particle distance determined by the MPA ligands led to mobilities up to 4 × 10^−4^ cm^2^·V^−1^·s^−1^. Furthermore, the doping/hole concentration (~2 × 10^15^ cm^−3^) was sufficiently low to yield very small dark currents, which is primordial for obtaining high photoconductive sensitivities and detectivities. In the case of TBAI, where the doping level was higher (~2 × 10^16^ cm^−3^), the charge transport seems to be influenced by an additional band associated with mid-gap trap states (QD aggregation may play a role in this mechanism), leading to much higher dark currents. The resulting effect is the smaller sensitivity and detectivity deduced for photodevices based on TBAI-treated devices. From the point of view of the kinetics associated with the excess photogenerated carriers, values for both MPA- and TBAI-treated QD-solids were not too different, as revealed by their behavior under relatively high photocarrier injection levels (10^14^–10^15^ cm^−3^). The major difference in the majority charge-carrier transport under dark conditions, as mentioned previously, limits the detectivity for very low light signals, which is regulated by relatively shallow traps close to the conduction band of the QD solid. These conclusions were validated through a model that included the presence of such “safe traps” that nicely reproduced the observed photocurrent and responsivity evolution with the incident light power. Values of responsivity as high as 15–70 A/W (for 20- and 5-μm channel lengths, respectively) at 1550 nm, were reached for MPA-treated photodevices, but only under very low incident powers, with a negative counterpart associated to the slow photo-response. The lower noise in these MPA-treated devices makes detectivity greater than 10^11^ (10^12^ if shot noise dominates) Jones. On the basis of our model, there are only two major routes for improving future photodevices based on QD solids depending on the photocarrier injection level: (1) for detection of very-low-light signals, the use of “safe traps” is positive in order to obtain very high photoconductive responsivity/gain, but it will be necessary to reduce the trap energy depth (i.e., create traps as shallow as possible) to reduce the response time; and (2) for detection of high-light signals, the only way of increasing the photoconductive gain is through an effective increase in carrier mobilities in the QD solid.

## Figures and Tables

**Table 1:** Conductivity under dark conditions (σ_0_), photoconductivity (**Δ**σ), and photoconductive sensitivity (S) of processed PbS quantum-dot (QD) photoconductors treated with 3-mercaptopropionic acid (MPA) and tetrabutylammonium iodide (TBAI) ligands, as deduced from I–V curves in Figure 3.

	MPA			TBAI		
Gap (μm)	σ_0_ * (μS/cm)	Δ*σ* (μS/cm)	*S* = *(I_L_ − I_D_)/I_L_*	σ_0_ * (μS/cm)	Δ*σ* (μS/cm)	*S* = *(I_L_ − I_D_)/I_L_*
2	0.20–0.37	8.5	0.97	1.0–4.0	6	0.82
5	0.33–0.58	5.3	0.93	0.9–2.6	4	0.76
20	0.03–0.05	0.95	0.93	0.58	0.20–0.65	0.2–0.4

* The I–V curves under dark conditions exhibited a near quadratic behavior on applied voltage, more evident above 4–5 V; hence, we included a range of variation for σ_0_, being the smallest/highest value corresponding to low- and high-bias-voltage regions; this effect was less important in the case of the photoconductor with electrodes separated 20 μm (weaker electric fields). In the case of **Δ***σ* deduced from *I_L_* − *I_D_* versus voltage, the variation was mostly linear, and the value was obtained with a relative error of around 10% (except for the photoconductor with the largest gap treated with TBAI, in which the range of variation is included).

**Table 2:** Acceptor concentration and mobility of processed PbS QD solids treated with MPA and TBAI ligands, as obtained from data shown in Figures S4 and S5. From these values, estimated conductivity and drift/transit time are also listed.

Electrical Parameter	MPA	TBAI
**Acceptor concentration, Na** (cm^−3^)	1.9 × 10^15^	2.3 × 10^16^
**Hole mobility, *μ_p_*** (cm^2^·V^−1^·s^−1^)	(1–4) × 10^−4^	(2–6) × 10^−5^
**Conductivity, *σ_0_ = q μ_p_ p* (μS/cm)**	0.03–0.12	0.07–0.22
**Drift/transit time, *τ_d_ = L^2^/μV*** (μs) ***L*** = 5 μm; ***V*** = 100 V	6–25	40–125

## Supplementary Materials

The following are available online at http://www.mdpi.com/2079-4991/8/9/677/s1, Figure S1: Cross sectional scanning electron microscopy (SEM) images of the PbS QD-solid deposited on a SiO_2_ substrate by the doctor blade method, Figure S2: Transmission electron microscopy image of PbS QDs with different surface ligands. From left to right: PbS-OAm; PbS-MPA; PbS-TBAI, Figure S3: Double logarithmic plot of the I-V curves (data symbols) mesured under dark conditions in the PbS-TBAI photoconductors. The best fit to the SCLC equation is obtained for *m* = 3.5. Coefficient *b* is smaller and practically negligible for the photoconductors with 5 and 20 m of channel length, respectively, Figure S4: Transfer FET curves of processed PbS colloidal QD photoconductors treated with MPA (red curve) and TBAI (green curve). The inset shows the QD-solid film deposited by doctor blade technique in three FET devices prior to treatement by TBAI and MPA, Figure S5: C–V characteristics of ITO/PEDOT/PbS/Ag Schottky devices with ligand exchange (a) PbS-MPA and (b) PbS-TBAI, Figure S6: Short circuit photocurrent densities plotted against open circuit voltages measured in the same device under AM1 conditions for different Schottky photodiodes based on MPA-treated QD-solids (300–600 nm thick), Figure S7: (a) Microscope photography of a 20 μm gap interdigitated device prior to the PbS QD nanoink deposition. (b) Photocurrent measured in the interdigitated device (red symbols) as compared to 10 (green symbols) and 20 (blue symbols) μm gap two-electrode photoconductors as a function of applied bias under AM1-solar illumination. (c) Responsivity in the interdigitated photoconductor device at several applied bias as a function of the incident power using a 1550 nm laser source (the responsivity is estimated by assuming that the whole laser beam is collected by the device), Table S1: Performances of different photoconductive detectors based on PbS nanocrystals (standard data for PbS bulk is also included).
