# Supplementary material for: Charge Transport in Trap-Sensitized Infrared PbS Quantum-Dot-Based Photoconductors: Pros and Cons
Source: Nanomaterials (Basel). 2018 Aug 30;8(9):677. doi: 10.3390/nano8090677 (PMC6165075; doi:10.3390/nano8090677)
Supplement: Supplementary file 1 [file nanomaterials-08-00677-s001.pdf]

nanomaterials

Effects of Passivation Strategy on Charge Dynamics and Figures of Merit of Infrared PbS Quantum-Dot-Based Photoconductors

Alberto Maulu, Juan Navarro-Arenas, Pedro J. Rodríguez-Cantó†, Juan F. Sánchez-Royo, Rafael Abargues, Isaac Suárez, Juan P. Martínez-Pastor

Supplementary Information /APPENDIX

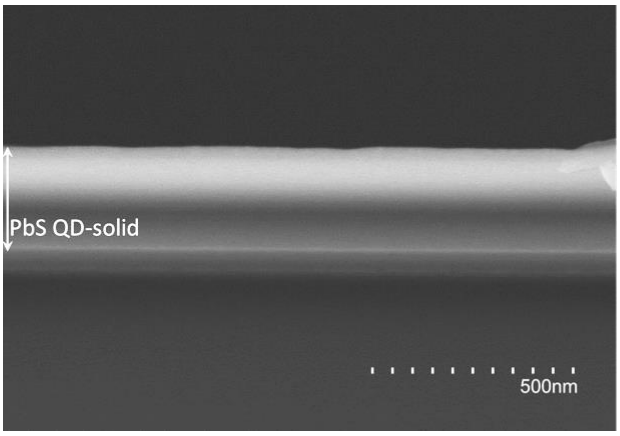

Figure S1. Cross-sectional scanning electron microscopy (SEM) images of the PbS QD solid deposited on the SiO_2 substrate using the doctor-blade method.

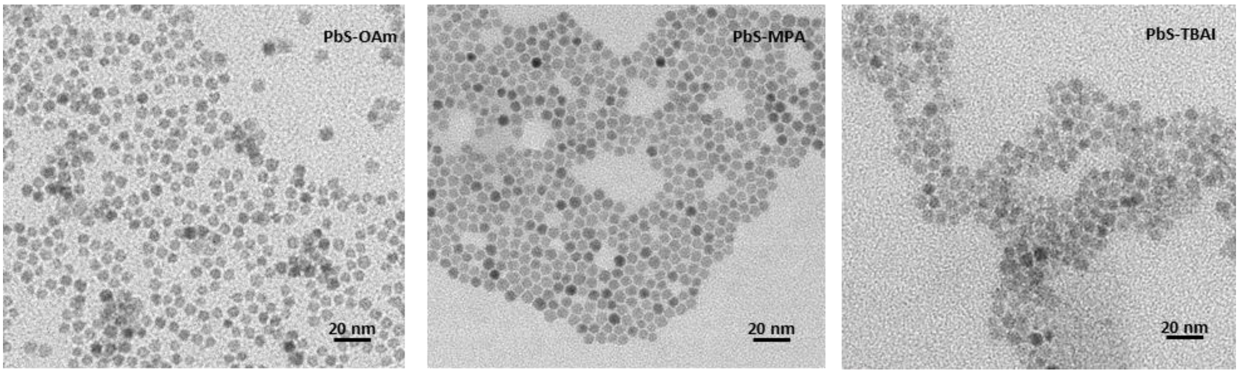

Figure S2. Transmission electron microscopy images of PbS QDs with different surface ligands. From left to right: PbS–OAm; PbS–MPA;PbS–TBAI.

The I–V curves measured under dark conditions exhibit a nonlinear behaviour, which is more evident in the TBAI-treated PbS QD solid. This nonlinear behaviour can be nicely fitted using the I–V response [1]:

$$I = aV + bV^m,$$

where a, b, and m are fitting paramenters. This response is due to the space-charge-limited current (SCLC) effect that ocurs when uncompensated charge carriers are injected into the material, as reported for Si-nanocrystal films [1]. Figure S3 shows this behaviour, and the best fit to the previous SCLC equation yields m = 3.5 for the I–V curves measured in photoconductors with 2- and 5-µm channel length (in the 20-mm device, the potential behaviour is practically absent in the measured voltage bias range), and only b is found to decrease with the channel length (b contains the channel length that defines the electric field inside the material). In the presence of traps, part of the injected

carriers can be considered as free, and exponent m may be greater than 2, as explained in Reference [1]. The I–V curves measured under dark conditions in MPA-treated PbS QD solids show a nonlinear behaviour which is less important in this voltage range; however, similar fits can be made using approximately the same values for m and smaller b-values (a factor three times smaller when comparing TBAI- and MPA-treated photoconductors with 2-μm channel length).

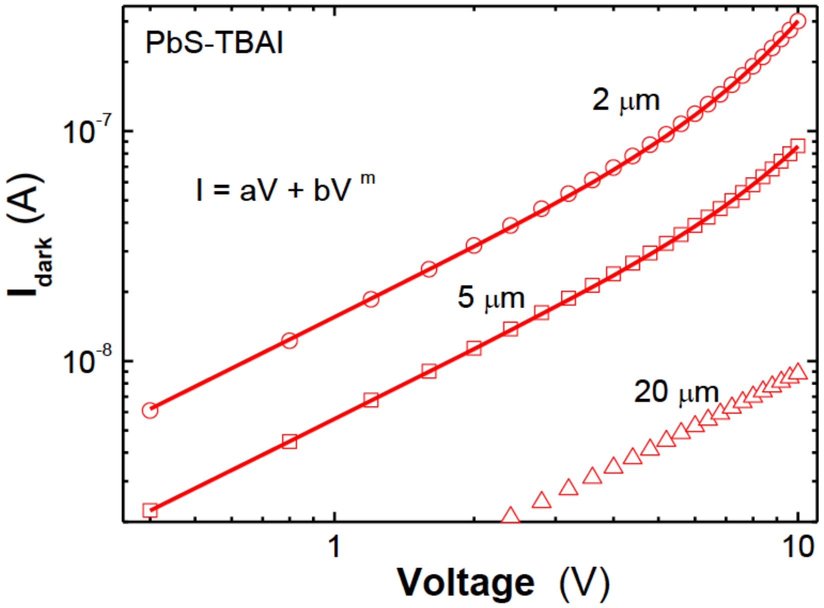

Figure S3. Double logarithmic plot of the I–V curves (data symbols) mesured under dark conditions in the PbS–TBAI photoconductors. The best fit to the space-charge-limited current (SCLC) equation is obtained for m = 3.5. Coefficient b is smaller and practically negligible for the photoconductors with 5- and 20-μm channel length, respectively.

The field-effect mobility measurements give important insight into how the passivation strategy influences the charge-carrier transport in the PbS QD solids, as shown in Figure S4. The deposition of the PbS QD nanoink on the prefabricated FET devices (from Ossila), also made using the doctor-blade method as described in Section 2.3, resulted in QD-solid formation after ligand exchange, as shown in the inset of Figure S4 (a similar area was deposited onto our fabricated chips).

The field-effect (FET) mobility in the linear regime was determined using the characteristic output curves of the field-effect transistor (FET), given by the plot of drain-source current (I_{DS}) versus gate voltage (V_{GS}) for a given drain voltage (V_{DS}) bias (Figure S4). The output curves can be divided into two regions: the linear region and the saturation region. The slope of the linear region can be used to obtain the charge-carrier mobility using the following equation, valid if charge-carrier mobility is assumed to be temperature- and field-independent:

$$\frac{\partial I_{DS}}{\partial V_{GS}} = \frac{W}{L} \mu C_S V_{DS},$$

where L is the channel length (results in Figure S3 correspond to 30 μm), W (1 mm) is the channel width, and the capacitance of the insulating layer C_{SiO2} of 300-nm-thick Ossila substrates is 1.15 × 10^{-8} F·cm^{-2}. The drain-source voltage was set at 1V. To ensure field independency, mobility was measured at different channel lengths. The mobility obtained for the PbS–MPA FET devices was around (1–4) × 10^{-4} cm^2·V^{-1}s^{-1}, whereas PbS–TBAI passivated films displayed a carrier mobility of (2–6) × 10^{-5} cm^2·V^{-1}s^{-1}, as represented by the linear fits in Figure S4 (continuous lines).

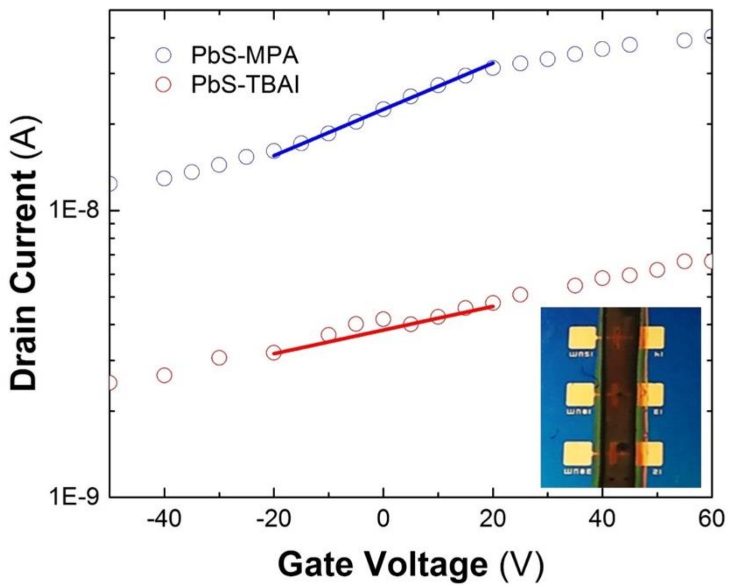

Figure S4. Transfer FET curves of processed PbS colloidal QD photoconductors treated with MPA (red curve) and TBAI (green curve). The inset shows the QD-solid film deposited with the doctor-blade technique in three FET devices prior to treatement with TBAI and MPA.

For PbS–TBAI QD solids, we found higher dark conductivity levels than in the case of PbS–MPA (**Error! Reference source not found.** of the manuscript). The electrical conductivity in a p-doped semiconductor is directly proportional to the hole mobility and the free-hole concentration. Above, we estimated carrier mobilities from FET devices, but they were different by a factor of five, being smaller for TBAI-treated QD solids; hence, the free-hole concentration should be significantly higher in this case with respect to MPA-treated films. From the slope of $C^{-2}(V)$ curves measured in Schottky-like diodes (glass/ITO/PEDOT/QD solid/Ag) (Figure S5), we can deduce the doping concentration for both TBAI- and MPA-treated PbS QD solids giving rise to the built-in Schottky barriers [2].

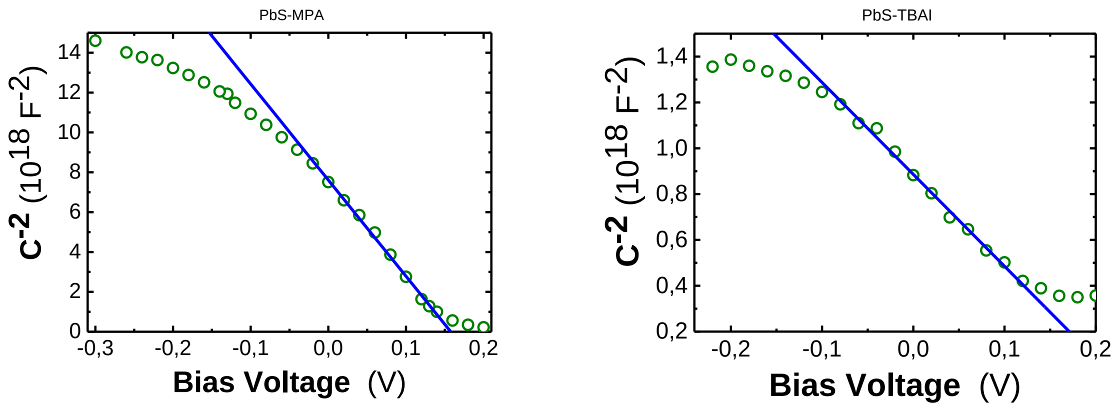

(**a**) (**b**)

Figure S5. C–V characteristics of ITO/PEDOT/PbS/Ag Schottky devices with ligand exchange: (**a**) PbS–MPA and (**b**) PbS–TBAI.

Figure S6 plots measured short-circuit density currents versus open-circuit voltage in several MPA-treated Schottky-heterojunction photodiodes under AM1 illumination conditions.

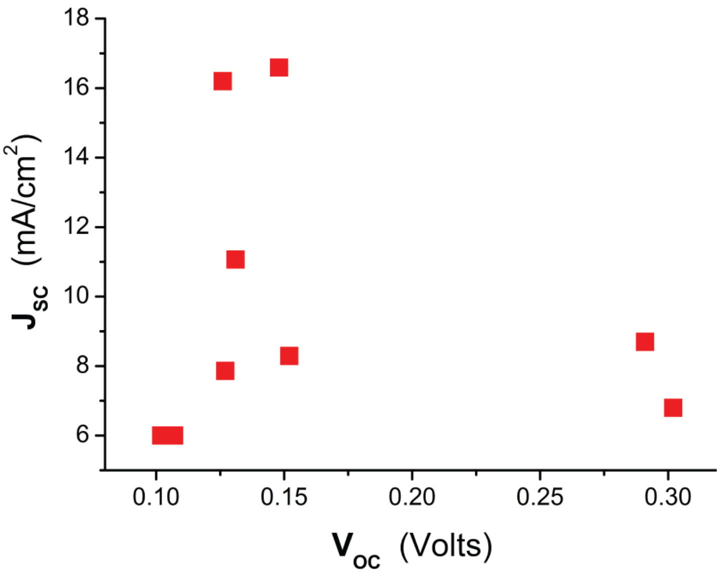

Figure S6. Short-circuit photocurrent densities plotted against open-circuit voltages measured in the same device under AM1 conditions for different Schottky photodiodes based on MPA-treated QD solids (300–600 nm thick).

Interdigitated photoconductor devices (as the one shown in Figure S7a) are an alternative to single two-electrode photoconductors or FET detectors, because, in the first case, smaller bias voltages are needed (a factor approximately equal to the number of metal finger pairs) to obtain similar photocurrents. Figure S7b shows the photocurrent as a function of the applied bias recorded under solar AM1 illumination (i.e., an incident density power of 100 mW/cm^2 that gives different collected light in the three devices because of their different active area: 200 and 20–10 µW in the 20-µm-interdigitated and 20–10-µm-gap photoconductors, respectively). As expected, the photocurrent measured in the interdigitated photoconductor at 10 V was similar (smaller) to that measured at 100 V in the 20- (10)-µm-gap photoconductor. The measured responsivity obtained in the interdigitated photodetector decreased with incident power (Figure S7c), as previously observed and discussed in the main text for two-electrode photoconductors (Figure 4c). The absolute value of responsivity in this interdigitated device, 7 mA/W at 10 V, is not far from the value reported in Reference [3] for the same finger gap (40 mA/W at 1 V, with an interdigitated device containing five times as many finger pairs).

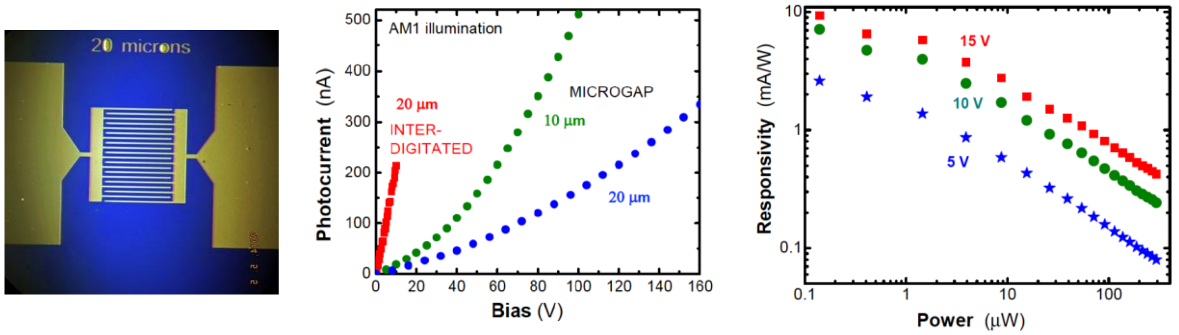

(**a**) (**b**) (**c**)

Figure S7. (**a**) Microscope photograph of a 20-µm-gap interdigitated device prior to the PbS QD nanoink deposition. (**b**) Photocurrent measured in the interdigitated device (red symbols) as compared to 10- (green symbols) and 20- (blue symbols) µm-gap two-electrode photoconductors as a function of applied bias under AM1 solar illumination. (c) Responsivity in the interdigitated photoconductor device at several applied bias voltages as a function of the incident power using a

1550-nm laser source (responsivity was estimated by assuming that the whole laser beam was collected by the device).

Table S1. Performances of different photoconductive detectors based on PbS nanocrystals (standard data for PbS bulk is also included).

**Photoactive Material (Ligand treatment)**	**Type**	**Spectra range (nm)**	**Responsivity**	**Detectivity (Jones)**	**Rise/Decay time and/or Bandwidth**	**Reference**
PbS bulk	Photoconductor	1000–3000	5×10^4 V·W^{-1} (0.1 A·W^{-1} for 500-k load)	1×10^{11}	-	[4]
PbS NC (butylamine)		800–1500	2700 A·W^{-1}	1.8×10^{13}	18 Hz	[4]
PbS NC (butylamine)	Photoconductor	400–900	113 A·W^{-1}	5×10^{12}	8 Hz	[4]
PbS NC (As_2S_3)	Photoconductor	900–1550	200 A·W^{-1}	1.2×10^{13}	8 Hz	[4]
PbS NC (OH^-/ S^{2-})	Photoconductor (interdigitated)	NIR <2400	50/8 A·W^{-1} (1550 nm, 230 K)	$(3.4/2.8) \times 10^8$	40 Hz	[4,5]
PbS NC Ag NP (EDT)	Photoconductor	400–1700	5 A·W^{-1}	2.5×10^{11}	200 Hz	[4]
PbS NC Ag NC (MPA)	Photoconductor	350–800	4 mA·W^{-1}	7.1×10^{10}	9.4 Hz	[4]
PbS NC PBCM (EDT)	Photoconductor	800–1400	57%	4.4×10^7	330 kHz	[4]
PbS NC MWCNTs (MPA)	Photoconductor	Visible–NIR	0.583 A·W^{-1}	3.2×10^{12}	-	[4]
PbS NC (Oleic)	Photoconductor (interdigitated)	850–1550	30 A·W^{-1}	2×10^{10}	160 ms/3 s<br>0.1 Hz	[3]
PbS NC (bilayer EDT/TBAI)	Photoconductor	450–1100	0.27 A·W^{-1}(580 nm)	1.7×10^{12}	3.6/30 ms<br>0.1 Hz	[6]
PbS/graphene (EDT)	Phototransistor	600–1600	10^7 A·W^{-1}	7×10^{13}	10 Hz	[5]
PbS/MoS_2 (EDT)	Phototransistor	500–1150	6×10^5 A·W^{-1}	5×10^{11}	-	[5]
PbS/WS_2	Phototransistor	Visible–NIR	14 A·W^{-1}	3.9×10^8	153/226 s	[7]
PbS/ZnO	Photoconductor	Ultraviolet–	0.051 A·W^{-1}	3.4×10^8	9/2 s	[8]
PbS/graphene	Phototransistor	-	420 A·W^{-1}	2.1×10^9	12/198 ms	[9]
Organic/PbS	Photoconductor	400–1000	6.32 A·W^{-1}	1.12×10^{13}	0.42/0.37 s	[10]
PbS NC (perovskite shells)	Photoconductor	850–1400	1.3 A·W^{-1}(1100 nm)	2×10^{11}	3.6/30 ms<br>110 kHz	[11]
PbS NC (MPA)	Photoconductor	950–1650	70 / 15 A W^{-1} (1550 nm, 5 / 20 m gap)	10^{12}/5×10^{12}<br><10^{11} (Eq. 6)	1/15 s	This work
PbS NC (TBAI)	Photoconductor	950–1650	0.3 / 0.1 A W^{-1} (1550 nm 5 / 20 m gap)	1.7×10^9/5×10^{10}	2/16 s	This work

NC = Nanocrystals; NP = nanoparticles; PBCM = conducting polymer, MWCNTs = multi-well carbon nanotubes.

References in Supplementary Information

1. Pereira, R. N.; Niesar, S.; You, W. B.; da Cunha, A. F.; Erhard, N.; Stegner, A.R.; Wiggers, H.; Willinger, M.G.; Stutzmann, M.; Brandt, M.S. Solution-processed networks of silicon nanocrystals: The role of internanocrystal medium on semiconducting behavior. *J. Phys. Chem. C.* **2011**, *115*, 20120–20127.
2. Maulu, A.; Rodríguez Cantó, P.J.; Navarro Arenas, J.; Abargues, R.; Sanchez-Royo, J.F.; García Calzada, R.; Martínez-Pastor, J.P. Strongly-coupled PbS QD solids by doctor blade for IR photodetection. *RSC Adv.* **2016**, *6*, 80201–80212.
3. De Iacovo, A.; Venettacci, C.; Colace, L.; Scopa, L.; Foglia, S. Noise performance of PbS colloidal quantum dot photodetectors, *Apl. Phys. Lett.* **2017**, *111*, 211104; *ibid*, PbS colloidal quantum dot photodetectors operating in the near infrared. *Sci. Rep.* **2016**, *6*, 37913.
4. Saran, R.; Curry, R.J. Lead sulphide nanocrystal photodetector technologies. *Nat. Photonics* **2016**, *10*, 81–92.
5. Hu, C.; Gassenq, A.; Justo, Y.; Devloo-Casier, K.; Chen, H.; Detavernier, C.; Hens, Z.; Roelkens, G. Air-stable short-wave infrared PbS colloidal quantum dot photoconductors passivated with Al_2O_3 atomic layer deposition. *Appl. Phys. Lett.* **2014**, *105*, 171110.
6. Ren, Z.; Sun, J.; Li, H.; Mao, P.; Wei, Y.; Zhong, X.; Hu, J.; Yang, S.; Wang, J. Bilayer PbS quantum dots for high-performance photodetectors. *Adv. Mater.* **2017**, *29*, 1702055.
7. Yu, Y.; Zhang, Y.; Song, X.; Zhang, H.; Cao, M.; Che, Y.; Dai, H.; Yang, J.; Zhang, H.; Yao, J. PbS-decorated WS_2 phototransistors with fast response. *ACS Photonics* **2017**, *4*, 950–956.
8. Zheng, Z.; Gan, L.; Zhang, J.; Zhuge, F.; Zhai, T. An enhanced UV–Vis–NIR and flexible photodetector based on electrospun ZnO nanowire array/PbS quantum dots film heterostructure. *Adv. Sci.* **2017**, *4*, 1600316.
9. Che, Y.; Zhang, Y. Cao, X. Song, X.; Zhang, H.; Cao, M.; Dai, H. Yang, J.; Zhang, G.; Yao, J. High-performance PbS quantum dot vertical field-effect phototransistor using graphene as a transparent electrode. *Appl. Phys. Lett.* **2016**, *109*, 263101.
10. Wei, Y.; Ren, Z.; Zhang, A.; Mao, P.; Li, H.; Zhong, X.; Li, W.; Yang, S.; Wang, J. Hybrid organic/PbS quantum dot bilayer photodetector with low dark current and high detectivity. *Adv. Funct. Mater.* **2018**, *28*, 1706690.
11. Sytnyk, M.; Yakunin, S.; Schöfberger, W.; Lechner, R.T.; Burian, M.; Ludescher, L.; Killilea, N.A.; YousefiAmin, A.; Kriegner, D.; Stangl, J.; et al. Quasi-epitaxial metal-halide perovskite ligand shells on PbS nanocrystals. *ACS Nano* **2017**, *11*, 1246–1256.

© 2018 by the authors. Submitted for possible open access publication under the terms and conditions of the Creative Commons Attribution (CC BY) license (http://creativecommons.org/licenses/by/4.0/).
